# Quininium tetra­chloridozinc(II)

**DOI:** 10.1107/S1600536809035442

**Published:** 2009-09-05

**Authors:** Li-Zhuang Chen

**Affiliations:** a Ordered Matter Science Research Center, College of Chemistry and Chemical Engineering, Southeast University, Nanjing 210096, People’s Republic of China

## Abstract

The asymmetric unit of the title compound {systematic name: 2-[hydr­oxy(6-meth­oxy­quinolin-1-ium-4-yl)meth­yl]-8-vinyl­quinuclidin-1-ium tetra­chlorido­zinc(II)}, (C_20_H_26_N_2_O_2_)[ZnCl_4_], consists of a double proton­ated quininium cation and a tetra­chloridozinc(II) anion. The Zn^II^ ion is in a slightly distorted tetra­hedral coordination environment. The crystal structure is stabilized by inter­molecular N—H⋯Cl and O—H⋯Cl hydrogen bonds.

## Related literature

For ferroelectric behavior, see: Fu *et al.* (2007 ▶, 2008*b* ▶). For non-linear optical second harmonic generation, see: Qu *et al.* (2003*b* ▶). For transition-metal complexes of quinine, see: Fu *et al.* (2008*a* ▶); Qu *et al.* (2003*a* ▶); Zhao *et al.* (2003 ▶).
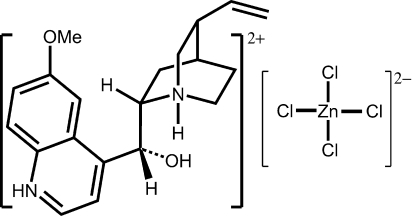


## Experimental

### 

#### Crystal data

(C_20_H_26_N_2_O_2_)[ZnCl_4_]
*M* *_r_* = 533.60
Orthorhombic, 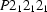

*a* = 9.518 (2) Å
*b* = 15.680 (5) Å
*c* = 15.846 (5) Å
*V* = 2364.8 (12) Å^3^
*Z* = 4
Mo *K*α radiation
μ = 1.51 mm^−1^
*T* = 293 K
0.30 × 0.28 × 0.26 mm

#### Data collection

Rigaku SCXmini CCD diffractometer
Absorption correction: multi-scan (*CrystalClear*; Rigaku, 2005 ▶) *T*_min_ = 0.660, *T*_max_ = 0.695
21856 measured reflections
4631 independent reflections
4325 reflections with *I* > 2σ(*I*)
*R*_int_ = 0.038

#### Refinement

*R*[*F* ^2^ > 2σ(*F* ^2^)] = 0.034
*wR*(*F* ^2^) = 0.087
*S* = 1.09
4631 reflections
267 parameters
H atoms treated by a mixture of independent and constrained refinement
Δρ_max_ = 0.83 e Å^−3^
Δρ_min_ = −0.39 e Å^−3^
Absolute structure: Flack (1983 ▶), 2005 Friedel pairs
Flack parameter: 0.007 (11)

### 

Data collection: *CrystalClear* (Rigaku, 2005 ▶); cell refinement: *CrystalClear*; data reduction: *CrystalClear*; program(s) used to solve structure: *SHELXS97* (Sheldrick, 2008 ▶); program(s) used to refine structure: *SHELXL97* (Sheldrick, 2008 ▶); molecular graphics: *SHELXTL* (Sheldrick, 2008 ▶); software used to prepare material for publication: *SHELXTL*.

## Supplementary Material

Crystal structure: contains datablocks I, global. DOI: 10.1107/S1600536809035442/hy2224sup1.cif

Structure factors: contains datablocks I. DOI: 10.1107/S1600536809035442/hy2224Isup2.hkl

Additional supplementary materials:  crystallographic information; 3D view; checkCIF report

## Figures and Tables

**Table 1:** Selected bond lengths (Å)

Cl1—Zn1	2.3097 (10)
Cl2—Zn1	2.3271 (10)
Cl3—Zn1	2.2701 (11)
Cl4—Zn1	2.2285 (11)

**Table 2:** Hydrogen-bond geometry (Å, °)

*D*—H⋯*A*	*D*—H	H⋯*A*	*D*⋯*A*	*D*—H⋯*A*
N1—H1⋯Cl1	1.01	2.17	3.167 (3)	169
N2—H2*A*⋯Cl2^i^	0.86	2.39	3.157 (3)	148
O2—H2*B*⋯Cl2	1.06 (4)	2.19 (4)	3.245 (3)	170 (3)

Symmetry code: (i) 
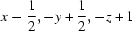
.

**Fig. 1.**
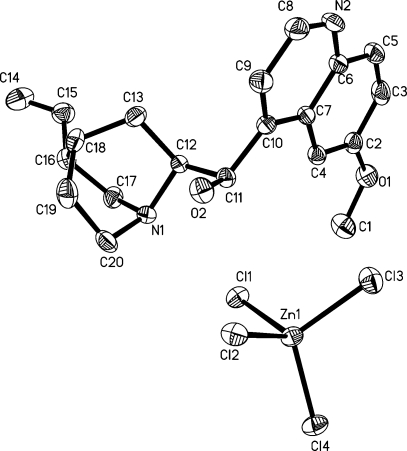
Molecular structure of the title compound. Displacement ellipsoids were drawn at the 30% probability level. H atoms have been omitted for clarity.

**Fig. 2.**
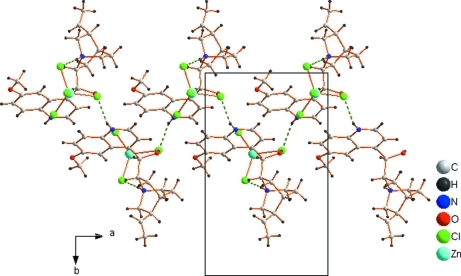
The packing diagram viewed along the c axis. Hydrogen bonds are drawn as dashed lines.

## supplementary crystallographic information

### Comment

The existence of a chiral centre in an organic ligand is very important for the construction of noncentrosymmetric or chiral coordination polymers that exhibit desirable physical properties, such as ferroelectricity (Fu *et al.*, 2007, 2008*b*) and nonlinear optical second harmonic generation (Qu *et al.*, 2003*b*). Quinine has a chiral centre, which has shown tremendous scope in the synthesis of transition-metal complexes (Fu *et al.*, 2008*a*; Qu *et al.*, 2003*a*; Zhao *et al.*, 2003). The construction of new members of this family of ligands is an important direction in the development of modern coordination chemistry. We report here the crystal structure of the title compound.

The asymmetric unit of the title compound consists of a double protonated quininium cation and a tetrachloridozinc anion (Fig. 1). The Zn^II^ ion is in a slightly distorted tetrahedral coordination environment (Table 1). Intermolecular N—H···Cl and O—H···Cl hydrogen bonds lead to a one-dimensional chain along the *a* axis (Table 2 and Fig. 2).

### Experimental

A mixture of quinine (0.324 g, 1 mmol), ZnCl_2_(0.136 g, 1 mmol) and 10% aqueous HCl (6 ml) were mixed and dissolved in 20 ml water by heating to 353 K (0.5 h), forming a clear solution. The reaction mixture was cooled slowly to room temperature and crystals of the title compound were formed after 5 d.

### Refinement

All H atoms were placed in calculated positions, except H1, H2B, H14A and H14B, and refined using a riding model, with C—H = 0.93–0.98 Å and N—H = 0.86 Å and with *U*_iso_(H) = 1.2(or 1.5 for methyl)*U*_eq_(C, N). H1, H2B, H14A and H14B were located in difference Fourier maps. The coordinates of H1 atom were fixed and the *U*_iso_(H1) parameter was refined. H2B atom was refined isotropically. H14A and H14B atoms were fixed with *U*_iso_(H) = 1.2*U*_eq_(C).

### Crystal data

**Table:**

(C_20_H_26_N_2_O_2_)[ZnCl_4_]	*F*(000) = 1096
*M**_r_* = 533.60	*D*_x_ = 1.499 Mg m^−^^3^
Orthorhombic, *P*2_1_2_1_2_1_	Mo *K*α radiation, λ = 0.71073 Å
Hall symbol: P 2ac 2ab	Cell parameters from 4325 reflections
*a* = 9.518 (2) Å	θ = 2.6–26.0°
*b* = 15.680 (5) Å	µ = 1.51 mm^−^^1^
*c* = 15.846 (5) Å	*T* = 293 K
*V* = 2364.8 (12) Å^3^	Block, colorless
*Z* = 4	0.30 × 0.28 × 0.26 mm

### Data collection

**Table:**

Rigaku SCXmini CCD diffractometer	4631 independent reflections
Radiation source: fine-focus sealed tube	4325 reflections with *I* > 2σ(*I*)
graphite	*R*_int_ = 0.038
Detector resolution: 13.6612 pixels mm^-1^	θ_max_ = 26.0°, θ_min_ = 2.6°
ω scans	*h* = −11→11
Absorption correction: multi-scan (*CrystalClear*; Rigaku, 2005)	*k* = −19→19
*T*_min_ = 0.660, *T*_max_ = 0.695	*l* = −19→19
21856 measured reflections	

### Refinement

**Table:**

Refinement on *F*^2^	Secondary atom site location: difference Fourier map
Least-squares matrix: full	Hydrogen site location: inferred from neighbouring sites
*R*[*F*^2^ > 2σ(*F*^2^)] = 0.034	H atoms treated by a mixture of independent and constrained refinement
*wR*(*F*^2^) = 0.087	*w* = 1/[σ^2^(*F*_o_^2^) + (0.0449*P*)^2^ + 0.0454*P*] where *P* = (*F*_o_^2^ + 2*F*_c_^2^)/3
*S* = 1.09	(Δ/σ)_max_ < 0.001
4631 reflections	Δρ_max_ = 0.83 e Å^−^^3^
267 parameters	Δρ_min_ = −0.39 e Å^−^^3^
0 restraints	Absolute structure: Flack (1983), 2005 Friedel pairs
Primary atom site location: structure-invariant direct methods	Flack parameter: 0.007 (11)

### Fractional atomic coordinates and isotropic or equivalent isotropic displacement parameters (Å^2^)

**Table:**

	*x*	*y*	*z*	*U*_iso_*/*U*_eq_	
C1	−0.0187 (4)	0.4580 (3)	0.6721 (3)	0.0622 (11)	
H1A	−0.0926	0.4733	0.7104	0.093*	
H1B	0.0314	0.5083	0.6553	0.093*	
H1C	0.0448	0.4192	0.6994	0.093*	
C2	0.0120 (3)	0.3916 (2)	0.5375 (2)	0.0439 (8)	
C3	−0.0535 (4)	0.3493 (3)	0.4687 (3)	0.0565 (10)	
H3A	−0.1510	0.3453	0.4672	0.068*	
C4	0.1544 (3)	0.4008 (2)	0.53945 (19)	0.0379 (7)	
H4A	0.1964	0.4303	0.5837	0.045*	
C5	0.0225 (4)	0.3151 (3)	0.4059 (3)	0.0546 (10)	
H5A	−0.0219	0.2884	0.3607	0.065*	
C6	0.1695 (4)	0.3200 (2)	0.4091 (2)	0.0439 (8)	
C7	0.2396 (3)	0.3655 (2)	0.47377 (19)	0.0360 (7)	
C8	0.3871 (5)	0.2812 (2)	0.3483 (2)	0.0522 (9)	
H8A	0.4358	0.2514	0.3069	0.063*	
C9	0.4602 (4)	0.3271 (2)	0.4088 (2)	0.0473 (8)	
H9A	0.5579	0.3290	0.4074	0.057*	
C10	0.3868 (4)	0.37042 (19)	0.47189 (19)	0.0370 (7)	
C11	0.4690 (3)	0.4239 (2)	0.5343 (2)	0.0359 (7)	
H11A	0.4266	0.4181	0.5903	0.043*	
C12	0.4575 (3)	0.51712 (19)	0.50559 (19)	0.0335 (6)	
H12A	0.3577	0.5282	0.4950	0.040*	
C13	0.5370 (4)	0.5386 (2)	0.4226 (2)	0.0450 (8)	
H13A	0.5877	0.4887	0.4030	0.054*	
H13B	0.4703	0.5550	0.3792	0.054*	
C14	0.5133 (5)	0.8073 (3)	0.3607 (3)	0.0702 (12)	
H14A	0.6090	0.8365	0.3835	0.084*	
H14B	0.4720	0.8325	0.3176	0.084*	
C15	0.4802 (5)	0.7337 (2)	0.3974 (2)	0.0543 (9)	
H15A	0.4002	0.7055	0.3785	0.065*	
C16	0.5622 (4)	0.6934 (2)	0.4666 (2)	0.0459 (8)	
H16A	0.6324	0.7345	0.4862	0.055*	
C17	0.4685 (4)	0.6691 (2)	0.5419 (2)	0.0466 (8)	
H17A	0.4823	0.7096	0.5874	0.056*	
H17B	0.3705	0.6711	0.5251	0.056*	
C18	0.6398 (3)	0.6114 (2)	0.4387 (2)	0.0463 (8)	
H18A	0.6929	0.6231	0.3869	0.056*	
C19	0.7406 (4)	0.5846 (3)	0.5081 (3)	0.0600 (10)	
H19A	0.8157	0.6262	0.5131	0.072*	
H19B	0.7820	0.5298	0.4944	0.072*	
C20	0.6600 (4)	0.5785 (2)	0.5913 (2)	0.0526 (9)	
H20A	0.6838	0.5258	0.6201	0.063*	
H20B	0.6849	0.6259	0.6277	0.063*	
N1	0.5046 (3)	0.58063 (17)	0.57192 (16)	0.0402 (6)	
H1	0.4592	0.5654	0.6278	0.054 (11)*	
N2	0.2491 (4)	0.27970 (18)	0.34941 (18)	0.0525 (8)	
H2A	0.2064	0.2518	0.3104	0.063*	
O1	−0.0779 (2)	0.41778 (18)	0.59904 (17)	0.0549 (6)	
O2	0.6096 (2)	0.39639 (15)	0.53783 (16)	0.0474 (5)	
H2B	0.623 (4)	0.386 (2)	0.604 (2)	0.050 (10)*	
Cl1	0.32557 (9)	0.53094 (5)	0.73304 (5)	0.0476 (2)	
Cl2	0.62582 (8)	0.38284 (6)	0.74196 (5)	0.0480 (2)	
Cl3	0.27211 (10)	0.29305 (6)	0.72517 (6)	0.0567 (2)	
Cl4	0.38062 (10)	0.39886 (6)	0.92702 (5)	0.0538 (2)	
Zn1	0.39476 (4)	0.40056 (2)	0.78665 (2)	0.04004 (11)	

### Atomic displacement parameters (Å^2^)

**Table:**

	*U*^11^	*U*^22^	*U*^33^	*U*^12^	*U*^13^	*U*^23^
C1	0.037 (2)	0.075 (3)	0.074 (3)	0.0023 (19)	0.0023 (18)	−0.016 (2)
C2	0.0350 (17)	0.0454 (19)	0.0514 (19)	−0.0047 (14)	−0.0067 (14)	0.0100 (17)
C3	0.038 (2)	0.060 (2)	0.071 (3)	−0.0166 (16)	−0.0170 (19)	0.013 (2)
C4	0.0322 (16)	0.0390 (16)	0.0424 (16)	−0.0033 (13)	−0.0059 (12)	0.0030 (15)
C5	0.057 (2)	0.058 (2)	0.049 (2)	−0.0198 (19)	−0.0221 (18)	0.0062 (19)
C6	0.056 (2)	0.0389 (17)	0.0373 (17)	−0.0115 (15)	−0.0109 (15)	0.0048 (14)
C7	0.0404 (18)	0.0328 (15)	0.0349 (16)	−0.0069 (13)	−0.0076 (13)	0.0028 (13)
C8	0.070 (3)	0.0444 (19)	0.0419 (19)	−0.0024 (18)	0.0033 (19)	−0.0030 (15)
C9	0.050 (2)	0.0464 (19)	0.0458 (19)	−0.0008 (15)	0.0029 (16)	−0.0048 (16)
C10	0.0435 (18)	0.0342 (15)	0.0332 (15)	−0.0023 (14)	−0.0046 (14)	0.0049 (12)
C11	0.0299 (15)	0.0419 (17)	0.0361 (16)	−0.0032 (12)	0.0003 (13)	−0.0015 (13)
C12	0.0322 (15)	0.0377 (16)	0.0305 (15)	−0.0063 (12)	−0.0002 (12)	−0.0013 (13)
C13	0.057 (2)	0.0420 (18)	0.0358 (18)	−0.0028 (15)	0.0082 (15)	0.0010 (15)
C14	0.101 (3)	0.057 (2)	0.052 (2)	0.012 (2)	0.008 (2)	0.002 (2)
C15	0.063 (2)	0.050 (2)	0.050 (2)	0.0002 (18)	−0.0042 (18)	0.0006 (18)
C16	0.051 (2)	0.0398 (18)	0.0471 (19)	−0.0092 (15)	−0.0009 (15)	−0.0010 (15)
C17	0.053 (2)	0.0382 (18)	0.048 (2)	−0.0040 (15)	0.0082 (16)	−0.0041 (16)
C18	0.0415 (19)	0.0481 (19)	0.0493 (19)	−0.0061 (14)	0.0130 (14)	0.0055 (16)
C19	0.036 (2)	0.062 (3)	0.082 (3)	−0.0065 (16)	0.0003 (18)	0.008 (2)
C20	0.050 (2)	0.052 (2)	0.056 (2)	−0.0138 (16)	−0.0220 (17)	0.0008 (17)
N1	0.0450 (15)	0.0444 (16)	0.0311 (14)	−0.0081 (12)	0.0002 (11)	−0.0049 (12)
N2	0.078 (2)	0.0424 (17)	0.0375 (15)	−0.0137 (15)	−0.0152 (15)	−0.0065 (13)
O1	0.0331 (13)	0.0688 (17)	0.0628 (16)	−0.0017 (11)	−0.0025 (11)	0.0023 (13)
O2	0.0338 (12)	0.0493 (13)	0.0592 (15)	0.0056 (11)	−0.0064 (10)	0.0012 (12)
Cl1	0.0530 (5)	0.0456 (4)	0.0441 (5)	0.0086 (4)	0.0062 (4)	0.0019 (4)
Cl2	0.0369 (4)	0.0630 (5)	0.0440 (4)	0.0088 (4)	0.0028 (3)	−0.0093 (4)
Cl3	0.0517 (5)	0.0551 (5)	0.0632 (6)	−0.0041 (4)	0.0097 (4)	−0.0149 (5)
Cl4	0.0589 (5)	0.0679 (5)	0.0345 (4)	0.0052 (5)	0.0070 (4)	0.0025 (4)
Zn1	0.0409 (2)	0.0449 (2)	0.03432 (18)	0.00593 (17)	0.00545 (15)	−0.00051 (16)

### Geometric parameters (Å, °)

**Table:**

C1—O1	1.433 (5)	C13—H13A	0.9700
C1—H1A	0.9600	C13—H13B	0.9700
C1—H1B	0.9600	C14—C15	1.330 (6)
C1—H1C	0.9600	C14—H14A	1.08
C2—O1	1.361 (4)	C14—H14B	0.88
C2—C4	1.364 (4)	C15—C16	1.486 (5)
C2—C3	1.419 (5)	C15—H15A	0.9300
C3—C5	1.343 (6)	C16—C17	1.537 (5)
C3—H3A	0.9300	C16—C18	1.546 (5)
C4—C7	1.431 (5)	C16—H16A	0.9800
C4—H4A	0.9300	C17—N1	1.506 (5)
C5—C6	1.402 (5)	C17—H17A	0.9700
C5—H5A	0.9300	C17—H17B	0.9700
C6—N2	1.367 (5)	C18—C19	1.519 (5)
C6—C7	1.416 (4)	C18—H18A	0.9800
C7—C10	1.404 (5)	C19—C20	1.529 (5)
C8—N2	1.314 (5)	C19—H19A	0.9700
C8—C9	1.386 (5)	C19—H19B	0.9700
C8—H8A	0.9300	C20—N1	1.511 (5)
C9—C10	1.396 (5)	C20—H20A	0.9700
C9—H9A	0.9300	C20—H20B	0.9700
C10—C11	1.514 (4)	N1—H1	1.01
C11—O2	1.407 (4)	N2—H2A	0.86
C11—C12	1.534 (4)	O2—H2B	1.06 (4)
C11—H11A	0.9800	Cl1—Zn1	2.3097 (10)
C12—N1	1.516 (4)	Cl2—Zn1	2.3271 (10)
C12—C13	1.554 (4)	Cl3—Zn1	2.2701 (11)
C12—H12A	0.9800	Cl4—Zn1	2.2285 (11)
C13—C18	1.525 (5)		
			
O1—C1—H1A	109.5	C15—C14—H14B	128.6
O1—C1—H1B	109.5	H14A—C14—H14B	116.4
H1A—C1—H1B	109.5	C14—C15—C16	124.6 (4)
O1—C1—H1C	109.5	C14—C15—H15A	117.7
H1A—C1—H1C	109.5	C16—C15—H15A	117.7
H1B—C1—H1C	109.5	C15—C16—C17	111.9 (3)
O1—C2—C4	125.2 (3)	C15—C16—C18	113.1 (3)
O1—C2—C3	114.5 (3)	C17—C16—C18	107.1 (3)
C4—C2—C3	120.2 (4)	C15—C16—H16A	108.2
C5—C3—C2	121.3 (4)	C17—C16—H16A	108.2
C5—C3—H3A	119.3	C18—C16—H16A	108.2
C2—C3—H3A	119.3	N1—C17—C16	109.9 (3)
C2—C4—C7	120.3 (3)	N1—C17—H17A	109.7
C2—C4—H4A	119.8	C16—C17—H17A	109.7
C7—C4—H4A	119.8	N1—C17—H17B	109.7
C3—C5—C6	119.2 (4)	C16—C17—H17B	109.7
C3—C5—H5A	120.4	H17A—C17—H17B	108.2
C6—C5—H5A	120.4	C19—C18—C13	108.6 (3)
N2—C6—C5	120.2 (3)	C19—C18—C16	108.9 (3)
N2—C6—C7	118.2 (3)	C13—C18—C16	111.3 (3)
C5—C6—C7	121.6 (4)	C19—C18—H18A	109.3
C10—C7—C6	118.9 (3)	C13—C18—H18A	109.3
C10—C7—C4	124.0 (3)	C16—C18—H18A	109.3
C6—C7—C4	117.1 (3)	C18—C19—C20	109.0 (3)
N2—C8—C9	120.2 (4)	C18—C19—H19A	109.9
N2—C8—H8A	119.9	C20—C19—H19A	109.9
C9—C8—H8A	119.9	C18—C19—H19B	109.9
C8—C9—C10	119.8 (4)	C20—C19—H19B	109.9
C8—C9—H9A	120.1	H19A—C19—H19B	108.3
C10—C9—H9A	120.1	N1—C20—C19	108.3 (3)
C9—C10—C7	119.2 (3)	N1—C20—H20A	110.0
C9—C10—C11	118.6 (3)	C19—C20—H20A	110.0
C7—C10—C11	122.2 (3)	N1—C20—H20B	110.0
O2—C11—C10	110.3 (3)	C19—C20—H20B	110.0
O2—C11—C12	111.8 (2)	H20A—C20—H20B	108.4
C10—C11—C12	107.3 (2)	C17—N1—C20	107.9 (3)
O2—C11—H11A	109.1	C17—N1—C12	108.6 (2)
C10—C11—H11A	109.1	C20—N1—C12	114.5 (3)
C12—C11—H11A	109.1	C17—N1—H1	113.3
N1—C12—C11	113.5 (2)	C20—N1—H1	103.6
N1—C12—C13	107.5 (2)	C12—N1—H1	108.9
C11—C12—C13	115.0 (3)	C8—N2—C6	123.7 (3)
N1—C12—H12A	106.8	C8—N2—H2A	118.1
C11—C12—H12A	106.8	C6—N2—H2A	118.1
C13—C12—H12A	106.8	C2—O1—C1	117.7 (3)
C18—C13—C12	109.4 (3)	C11—O2—H2B	101 (2)
C18—C13—H13A	109.8	Cl4—Zn1—Cl3	112.85 (4)
C12—C13—H13A	109.8	Cl4—Zn1—Cl1	111.13 (4)
C18—C13—H13B	109.8	Cl3—Zn1—Cl1	110.66 (4)
C12—C13—H13B	109.8	Cl4—Zn1—Cl2	111.06 (4)
H13A—C13—H13B	108.2	Cl3—Zn1—Cl2	105.47 (4)
C15—C14—H14A	114.8	Cl1—Zn1—Cl2	105.26 (4)

### Hydrogen-bond geometry (Å, °)

**Table:**

*D*—H···*A*	*D*—H	H···*A*	*D*···*A*	*D*—H···*A*
N1—H1···Cl1	1.01	2.17	3.167 (3)	169
N2—H2A···Cl2^i^	0.86	2.39	3.157 (3)	148
O2—H2B···Cl2	1.06 (4)	2.19 (4)	3.245 (3)	170 (3)

Symmetry codes: (i) *x*−1/2, −*y*+1/2, −*z*+1.
